# Expression, Prognostic Value, and Functional Mechanism of the KDM5 Family in Pancreatic Cancer

**DOI:** 10.3389/fcell.2022.887385

**Published:** 2022-04-13

**Authors:** Yunjie Duan, Yongxing Du, Zongting Gu, Xiaohao Zheng, Chengfeng Wang

**Affiliations:** State Key Lab of Molecular Oncology and Department of Pancreatic and Gastric Surgery, National Cancer Center/National Clinical Research Center for Cancer/Cancer Hospital, Chinese Academy of Medical Sciences and Peking Union Medical College, Beijing, China

**Keywords:** pancreatic cancer, prognostic markers, KDM5 family, bioinformatics analysis, pathogenesis introduction

## Abstract

**Background:** The histone lysine demethylase KDM5 family is an important epigenetic state-modifying enzyme family. Increasing evidence supports that epigenetic abnormalities in the KDM5 family are related to multiple cancers in humans. However, the role of the KDM5 family in pancreatic cancer is not clear, and related research is very scarce.

**Methods:** R software, Kaplan–Meier Plotter, cBioPortal, TIMER, LinkedOmics, STRING, Metascape, TISIDB, and the GSCA Lite online tool were utilized for bioinformatics analysis.

**Results:** KDM5A/B/C was significantly overexpressed in many kinds of tumor tissues, including pancreatic adenocarcinoma (PAAD), while the expression of KDM5D was significantly downregulated. The high expression of KDM5A/B/C was related to poor clinical features, such as worse treatment efficacy, higher tumor grade, and more advanced clinical stage. Patients with a family history of breast cancer and melanoma, history of drinking or history chronic pancreatitis were more likely to have KDM5A/B/C gene abnormalities, which were related to a variety of adverse clinical features. The results of gene ontology (GO) and kyoto encyclopedia of genes and genomes (KEGG) pathway analyses of the KDM5 family and its 800 co-expressed genes showed that many gene terms related to cell proliferation, migration and many carcinogenic pathways. Notably, we found that the expression level of KDM5A/B/C was positively correlated with the expression of multiple key driver genes such as KRAS, BRCA1, and BRCA2 etc. In addition, PPI network analysis showed KDM5 family proteins have strong interactions with histone deacetylase family 1 (HDAC1), which could modify the lysines of histone H3, and co-act on many pathways, including the “longevity-regulating pathway” and “Notch signaling pathway”. Moreover, the upregulation of KDM5A/B/C expression was associated with an increase in the infiltration of B cells, CD8^+^ T cells and other infiltrating immune lymphocytes and the expression levels of immune molecules such as NT5E and CD274. Interestingly, the overexpression of KDM5A/C was also corelated with reduced sensitivity of pancreatic cancer cells to many kinds of pancreatic cancer-targeting or chemotherapeutic drugs, including axitinib and gemcitabine.

**Conclusion:** KDM5 family members may be prognostic markers and new therapeutic targets for patients with pancreatic cancer.

## Introduction

Pancreatic cancer has attracted wide attention because of its unusually high mortality rate, and pancreatic cancer ranks fourth or fifth among causes of cancer-related death in most developed countries. At the beginning of the 21st century, the estimated number of pancreatic cancer cases in the world was 1,10,000, and the global mortality rate was estimated at 98% (Parkin et al., 2001). In 2021, an estimated 60,430 people will be diagnosed with pancreatic cancer in the United States alone, and approximately 48,220 are expected to die from the disease (Siegel et al., 2021). Currently, pancreatic cancer is the fourth most common cause of cancer-related death in men (after lung, prostate and colorectal cancer) and women (after lung, breast and colorectal cancer) in the United States (Siegel et al., 2021). Surgery and adjuvant therapy are cornerstones of the treatment of pancreatic cancer. Resection does lead to a 5-years survival rate of approximately 20%, but only 10% of patients can undergo pancreatic cancer resection due to the presence of widespread locally advanced lesions or metastases (Raimondi et al., 2009). However, even after radical resection, most patients experience relapse. Multimodal therapy based on a combination of neoadjuvant therapy, chemotherapy, radiotherapy, immunotherapy, and surgery seems to be an important strategy for improving prognosis (Neoptolemos et al., 2018), but the vast majority of pancreatic cancer patients are treated with current systemic chemotherapy regimens (FOLFIRINOX: 5-fluorouracil, leucovorin, irinotecan, and oxaliplatin; GNP: gemcitabine and nab paclitaxel). Chemotherapy resistance is an important factor affecting the efficacy of multidisciplinary therapy (Von Hoff et al., 2013), and thus, the prognosis of pancreatic cancer remains poor. Therefore, there is an urgent need to clarify the specific mechanisms of the occurrence and development of pancreatic cancer and chemotherapy resistance, develop new targeted drugs and prognostic markers, improve the efficacy of multidisciplinary comprehensive treatment, and prolong the survival time of patients with pancreatic cancer.

Stable inheritance of epigenetic state is essential for maintaining the specific functions of tissue and cell types. Previous studies have shown that epigenetic aberrations play an important role in the occurrence and development of tumors (Feinberg et al., 2006). Research on the function of epigenetic state-modifying enzymes has become a hot topic in tumor therapy. In eukaryotes, deoxyribonucleic acid (DNA) is packaged in the form of chromatin (Kornberg, 1974). The basic component of chromatin is the nucleosome, which consists of 146 bp of DNA wrapped on octamers of the four core histones (H2A, H2B, H3, and H4) (Luger et al., 1997). Histone tails are subjected to a variety of posttranslational modifications, including acetylation, methylation, phosphorylation, ubiquitin and SUMOylation (Shilatifard, 2006) which affect chromatin structure, thus affecting gene expression and DNA repair. Abnormal histone demethylation can lead to excessive cell proliferation, which leads to tumorigenesis and has been shown to be associated with many cancers (Cao et al., 2002; Milne et al., 2002; Nakamura et al., 2002; Yokoyama et al., 2004). The KDM5 family of histone lysine demethylases is an important family of epigenetic state-modifying enzymes that contain five conserved domains: JmjN, ARID, JmjC, PhD, and a C5HC2 zinc finger (Blair et al., 2011) and can specifically catalyze the trimethylation, dimethylation and monomethylated lysine 4 demethylation of histone H3, thus playing a central role in histone coding (Zhang et al., 2014; Johansson et al., 2016; Tumber et al., 2017). The family includes lysine demethylase 5A (KDM5A), lysine demethylase 5B (KDM5B), lysine demethylase 5C (KDM5C), and lysine demethylase 5D (KDM5D). Many studies have reported the role of the KDM5 family in the occurrence and development of many cancers. KDM5A is closely related to breast cancer, prostate cancer, ovarian cancer and small-cell lung cancer (Hou et al., 2012; Feng et al., 2017; Oser et al., 2019; Du et al., 2020). The expression of KDM5B promotes the invasiveness of non-small-cell lung cancer (NSCLC) (Kuo et al., 2018). Furthermore, increased expression of KDM5B promotes the growth of liver cancer cells and maintains chronic myeloid leukemia through multiple epigenetic effects (Gong et al., 2018; Xue et al., 2020). KDM5C is overexpressed in prostate cancer. It is a prognostic marker of prostate-specific antigen recurrence after radical prostatectomy (Stein et al., 2014). In contrast, KDM5D inhibits the invasion and metastasis of prostate cancer, and its overexpression can reduce the invasive ability of gastric cancer (Li et al., 2016; Gu and Chu, 2021). However, the role of the KDM5 family in pancreatic cancer is not clear, and related research is very scarce.

As such, this study analyzed data related to KDM5 family members in pancreatic cancer. First, we compared KDM5 family member expression and prognosis between pancreatic cancer samples and matched normal pancreatic tissues with The Cancer Genome Atlas (TCGA) and Genotype-Tissue Expression (GTEx) databases and further studied the possible mechanism by which the family members participate in the occurrence and development of pancreatic cancer through gene mutation, protein interaction, functional enrichment, and immune infiltration analyses. In addition, we used different databases to verify the results. The findings of this study will help to identify potential diagnostic markers and new treatment targets and ultimately improve the efficacy of multimodal comprehensive treatment of pancreatic cancer.

## Materials and Methods

### Ethical Statement

This study was approved by the Academic Committee of Cancer Hospital of Chinese Academy of Medical Sciences and carried out in strict accordance with the principles of the Helsinki Declaration. The data in this study were retrieved from online databases, all necessary written informed consent forms were obtained, and no human or animal experiments were performed.

### Expression Analysis

In this study, R (version3.6.3) was used to analyze the expression level (https://xenabrowser.net/datapages/) of the KDM5 family in tumor and paracancerous tissues in the PAAD TCGA cohort and normal pancreatic tissue in the GTEx database, and visualized with ggplot2 (3.3.3). The statistical significance of the differential expression was evaluated by the Wilcoxon test. To analyze the correlation between the gene expression levels of KDM5 family members and clinical variables, we used the R (version 3.6.3) to analyze PAAD samples from the TCGA database (https://portal.gdc.cancer.gov/). The statistical significance of the differential expression was evaluated by Fisher’s test.

### Gene Mutation Analysis

We used cBioPortal (https://www.cbioportal.org/) (Gao et al., 2013) to analyze the gene mutation of KDM5 family members in pancreatic cancer and further determined the correlation of these mutations with important clinicopathological factors. The statistical significance of the difference was evaluated by the chi-squared test. Furthermore, the log-rank test was used to evaluate the relationship between KDM5 family gene mutations and overall survival (OS) and disease-free survival (DFS) in patients with pancreatic cancer.

### Survival Analysis

We used Kaplan–Meier Plotter (http://kmplot.com/analysis/) (Győrffy et al., 2013) to analyze the correlation between the expression of KDM5 family genes and OS and relapse-free survival (RFS) in pancreatic cancer. The cutoff for low expression versus high expression was set as the value automatically selected by the best cutoff model, and the array with deviation was excluded. The log rank test was used to calculate the *p* value (*p* < 0.05).

### Correlation and Interaction Analyses

We used TIMER (http://timer.cistrome.org/) (Li et al., 2017) to analyze the correlations of KDM5 family gene expression in pancreatic cancer, and the statistical significance was evaluated by Spearman’s test. LinkedOmics (http://www.linkedomics.org/) (Vasaikar et al., 2018) was used to assess and draw a volcano plot of the correlations between KDM5 family members and 800 coexpressed genes in pancreatic cancer and a heatmap of the top 50 positively/negatively related genes. The genes with the strongest interaction with KDM5 family proteins were determined by combined score analysis via STRING (https://string-db.org/) (Szklarczyk et al., 2015), and the corresponding protein-protein interaction (PPI) network was constructed by STRING.

### Gene Annotation

The first 10 motifs with positive and negative correlations with the expression of each member of the KDM5 family were functionally annotated (https://www.genecards.org/) by GeneCards.

### Functional Enrichment Analysis

We used Metascape (https://metascape.org) (Zhou et al., 2019) to visualize the enriched biological process (BP), cellular composition (CC), molecular function (MF) and KEGG pathway terms of the KDM5 family and its coexpressed genes. Furthermore, the R (version 3.6.3) and clusterProfiler package (version 3.14.3) were used to visualize the enriched BP, CC, MF, and KEGG pathway terms of the 9 genes with the strongest interaction with KDM5 family proteins.

### Immune Infiltration Analysis

We used TIMER to analyze the correlation between the expression level of the KDM5 family and the degree of lymphocyte infiltration. The statistical significance of the difference was evaluated by Spearman’s test. In addition, TISIDB (http://cis.hku.hk/TISIDB/) (Ru et al., 2019) was also used to analyze the correlation between KDM5 family expression and the expression of immune molecules in pancreatic cancer. The difference was evaluated by Spearman’s test. We used the GSCA Lite online tool (http://bioinfo.life.hust.edu.cn/web/GSCALite/) (Liu et al., 2018) to analyze the correlation between KDM5 family expression and sensitivity to current chemotherapeutic or targeted drugs for pancreatic cancer. The difference was evaluated by Spearman’s test.

## Results

### Abnormal Expression of the KDM5 Family in Pancancer and PAAD Patients

**FIGURE 1:**
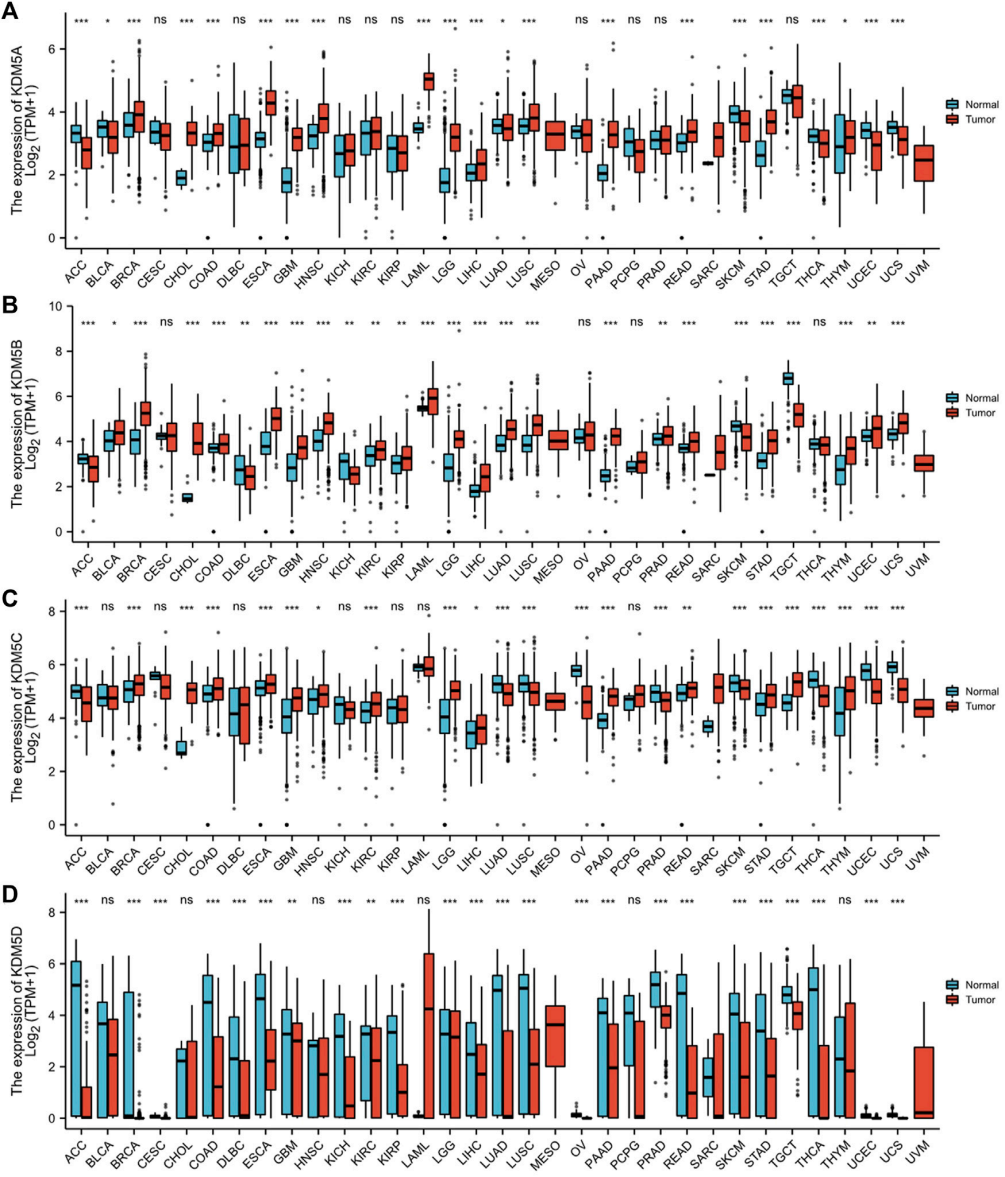
The expression level of the KDM5 family in different tumor tissues and paracancerous tissues in the TCGA database and normal tissues in the GTEx database. **(A)** The expression level of KDM5A; **(B)** The expression level of KDM5B; **(C)** The expression level of KDM5C; **(D)**The expression level of KDM5D. ns, *p* ≥ 0.05; *, *p* < 0.05; **, *p* < 0.01; ***, *p* < 0.001.

We used the R (version 3.6.3) to analyze the expression level of the KDM5 family in tumor and paracancerous tissues in TCGA and normal tissues in GTEx. The analysis of gene expression levels showed that KDM5 family genes were upregulated or downregulated in tumors compared with normal or paracancerous tissues in each type of cancer. Compared with that in paracancerous tissues or normal tissues, the expression of KDM5A/B/C was significantly increased in 12 kinds of tumor tissues, such as PAAD, breast invasive carcinoma (BRCA) and stomach adenocarcinoma (STAD), but significantly downregulated in only adrenocortical carcinoma (ACC) and skin cutaneous melanoma (SKCM) (Figures 1A–C). In contrast, the expression of KDM5D was significantly decreased in 24 kinds of tumor tissues, such as PAAD, BRCA, and prostate adenocarcinoma (PRAD) (Figure 1D). In summary, our results show that KDM5A/B/C is significantly overexpressed in a variety of tumor tissues, including PAAD, while the expression of KDM5D is significantly downregulated, indicating that the expression of KDM5 family members is closely related to the occurrence and development of many kinds of human malignant tumors, including pancreatic cancer.

### KDM5 Family Mutations and Their Correlation With Clinicopathological and Prognostic Features in Patients With PAAD

To further explore the mechanism of differential expression of the KDM5 family in pancreatic cancer, we used the cBioPortal online tool to analyze the gene mutations of the KD

**FIGURE 2:**
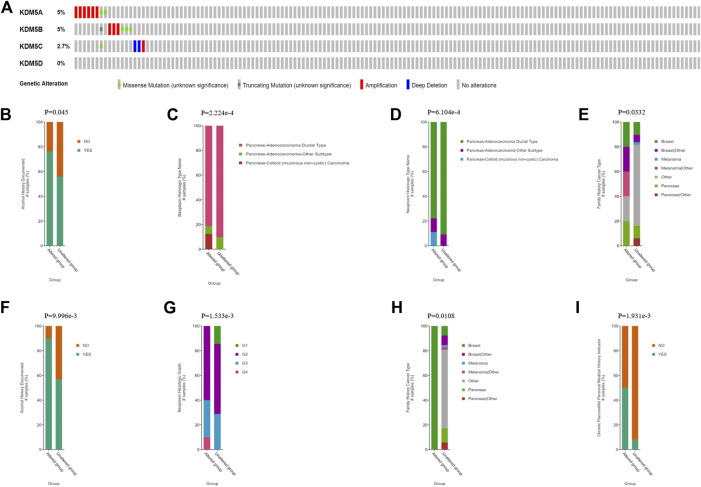
KDM5 family gene mutations and clinical and prognostic characteristics in patients with PAAD. **(A)** KDM5 family mutations in pancreatic cancer; **(B)** Pancreatic cancer patients with a history of drinking are more likely to undergo KDM5 family gene mutation; **(C)** Pancreatic mucinous adenocarcinoma patients are more likely to undergo KDM5 family genes mutation; **(D)** Pancreatic cancer patients with a family history of breast cancer and melanoma are more likely to undergo KDM5A gene mutation; **(E)** Pancreatic mucinous adenocarcinoma patients are more likely to undergo KDM5A gene mutation; **(F)** Pancreatic cancer patients with a history of drinking are more likely to undergo KDM5B gene mutation; **(G)** Pancreatic cancer patients with a family history of breast cancer are more likely to undergo KDM5C gene mutation; **(H)** Pancreatic cancer patients with a history of chronic pancreatitis are more likely to undergo KDM5C gene mutation; **(I)** Pancreatic cancer patients with a higher pancreatic cancer tumor grade are more likely to undergo KDM5C gene mutation.

M5 family. The KDM5 family had two or more types of gene variants in 17 samples (11%) from pancreatic cancer patients (Figure 2A). The most common variant was in the KDM5A/B gene (5%). The main types of variants were amplification and missense mutations. In addition, 2.7% of the variants occurred in KDM5C, and the most common type of variant was deep deletion, while no related mutations were detected in the KDM5D gene. Then, we comprehensively analyzed the clinical and pathological features of patients with KDM5 family gene mutations and those without mutations. The results showed that patients with a history of drinking and pancreatic cancer with mucinous adenocarcinoma were more likely to develop KDM5 family gene mutation (Figures 2B,C). Next, we analyzed the independent relationship between KDM5A/B/C gene mutation and clinicopathological features. The results showed that most of the patients with KDM5A gene mutation had a family history of breast cancer and melanoma or a pathological pancreatic cancer subtype of pancreatic mucinous adenocarcinoma (Figures 2D,E). Most of the patients with KDM5B gene mutation had a history of drinking (Figure 2F), and most of the patients with KDM5C gene mutation had a family history of breast cancer, a history of chronic pancreatitis and a higher pancreatic cancer tumor grade (Figures 2G–I). In the analysis of the prognostic characteristics of patients with KDM5 family gene mutation and those without mutation, it was not found that KDM5 family gene mutation had a significant effect on OS (Supplementary Figure S1A) and DFS (Supplementary Figure S1B) in patients with pancreatic cancer, which may have been related to the small sample size of patients. In summary, the frequency of KDM5A/B/C gene mutations was higher in patients with pancreatic cancer, while patients with a history of drinking, a family history of breast cancer, and a family history of melanoma or a history of chronic pancreatitis were more likely to develop KDM5A/B/C mutations. These mutations may lead to more malignant and higher-grade tumors. However, the effect of KDM5A/B/C gene mutation on the prognosis of patients with pancreatic cancer needs to be further clarified in studies with a large sample size.

### Correlation Between KDM5 Family Gene Expression and Clinical and Prognostic Characteristics in Patients With PAAD

**FIGURE 3:**
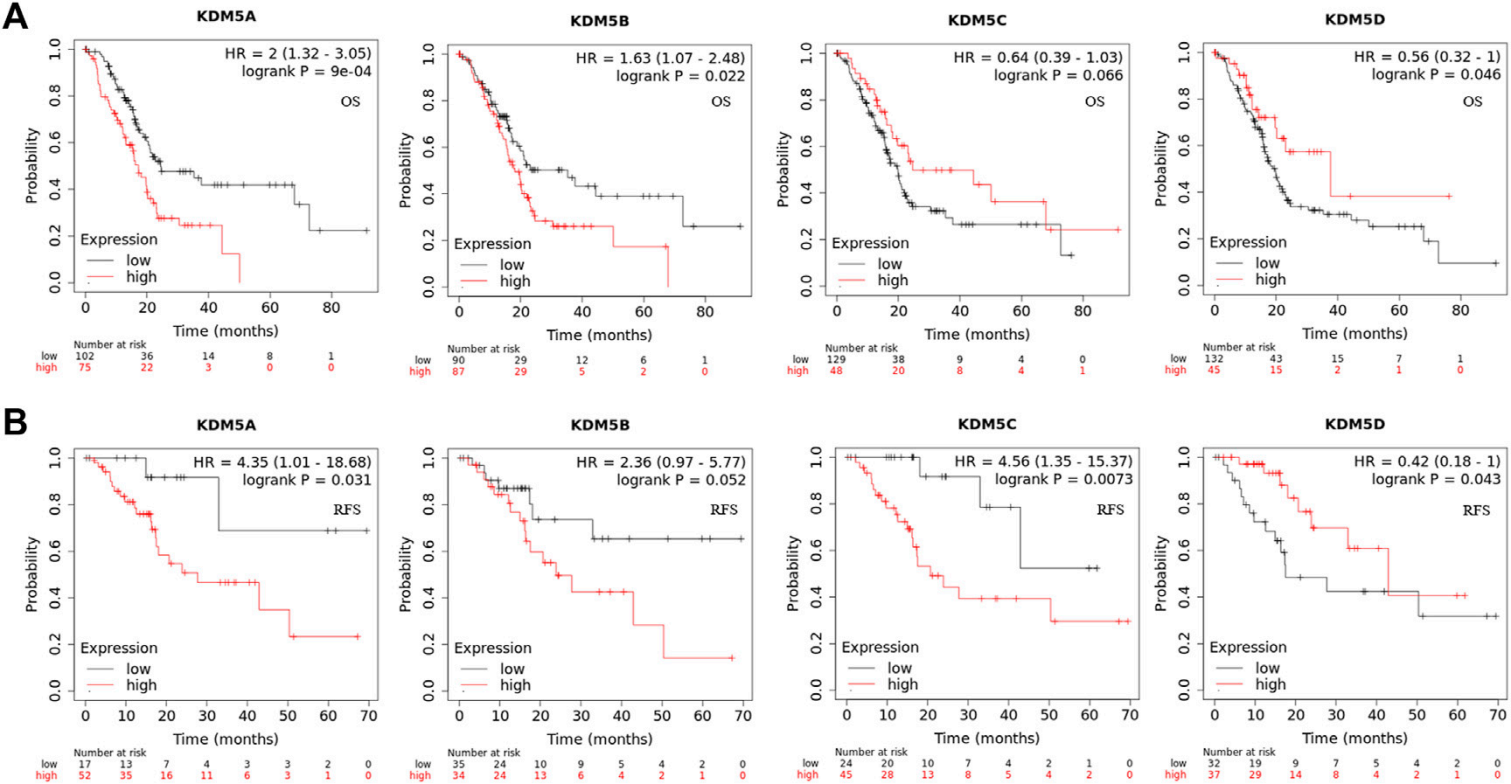
Gene expression level of and prognostic characteristics associated with the KDM5 family in patients with PAAD. **(A)** Upregulated expression of KDM5A is associated with shorter OS/Downregulation of KDM5D expression is significantly correlated with shorter OS; **(B)** Upregulated expression of KDM5A is associated with shorter RFS/Downregulation of KDM5D expression is significantly correlated with shorter RFS.

To analyze the correlation between the gene expression levels of KDM5 family members and clinical variables, we used the basic R package (version 3.6.3) to analyze the samples of the PAAD cohort in the TCGA database. As shown in Table 1, high expression of KDM5A was significantly associated with poorer treatment efficacy (*p* = 0.037) and higher tumor grade (*p* = 0.019). High expression of KDM5B was significantly correlated with age ≤65 years old (*p* = 0.036), no history of diabetes (*p* = 0.038), higher tumor grade (*p* = 0.048), more advanced clinical stage (*p* = 0.004), and more advanced T stage (*p* = 0.020). High expression of KDM5C was significantly correlated with female sex (*p* < 0.001). High expression of KDM5D was significantly correlated with male sex (*p* < 0.001) and smoking history (*p* = 0.041). The results of this study suggest that the high expression levels of KDM5A and KDM5B can increase the degree of malignancy of PAAD and lead to a worse prognosis of patients, further confirming that members of KDM5 family may be oncogenes of PAAD. Next, we analyzed the correlation between the expression levels of KDM5 family genes and prognosis in patients with pancreatic cancer via Kaplan–-Meier Plotter. The results showed that upregulated expression of KDM5A was associated with shorter OS and RFS (Figures 3A,B). Downregulation of KDM5D expression was significantly correlated with shorter OS and RFS (Figures 3A,B). In addition, although there was a lack of consistent significance in the associations with OS and RFS, overexpression of KDM5B and KDM5C was significantly associated with shorter OS and shorter RFS, respectively (Figures 3A,B). The above data show that abnormal expression of the KDM5 family can be used as a biomarker to predict the prognosis of patients with pancreatic cancer, which is worthy of further experimental data verification.

**TABLE 1:** Relationship Between KDM5 family member expression and clinicopathological features in the PAAD cohort.

Variable	Number (%) (*n* = 178)	KDM5A expression		*p*-value	KDM5B expression		*p*-value	KDM5C expression		*p*-value	KDM5D expression		*p*-value
		High (%) (*n* = 89)	Low (%) (*n* = 89)		High (%) (*n* = 89)	Low (%) (*n* = 89)		High (%) (*n* = 89)	Low (%) (*n* = 89)		High (%) (*n* = 89)	Low (%) (*n* = 89)	
Age (years) ≤65 > 65	93 (52.25)	51 (57.30)	42 (47.19)	0.230	54 (60.67)	39 (43.82)	0.036*	53 (59.55)	40 (44.94)	0.072	43 (48.31)	50 (56.18)	0.368
	85 (47.75)	38 (42.70)	47 (52.81)		35 (39.33)	50 (56.18)		36 (40.45)	49 (55.06)		46 (51.69)	39 (43.82)	
Gender	80 (44.94)	35 (39.33)	45 (50.56)	0.175	34 (38.20)	46 (51.69)	0.097	61 (68.54)	19 (21.35)	<0.001*	0 (0.00)	80 (89.89)	<0.001*
Female													
Male	98 (55.06)	54 (60.67)	44 (49.44)		55 (61.80)	43 (48.31)		28 (31.46)	70 (78.65)		89 (1.00)	9 (10.11)	
Race	11 (6.18)	6 (6.74)	8 (8.99)	0.685	9 (10.11)	3 (3.37)	0.057	8 (8.99)	4 (4.49)	0.412	4 (4.49)	8 (8.99)	0.580
Asian	6 (3.37)	4 (4.49)	3 (3.37)		6 (6.74)	1 (1.12)		5 (5.62)	2 (2.25)		3 (3.37)	4 (4.49)	
Black or African American	161 (90.45)	79 (88.76)	78 (87.64)		74 (83.15)	85 (95.51)		76 (85.39)	83 (93.26)		82 (92.13)	77 (86.52)	
White													
Smoker	82 (46.07)	36 (40.45)	46 (51.69)	0.149	35 (39.33)	47 (52.81)	0.088	43 (48.31)	39 (43.82)	0.627	34 (38.20)	48 (53.93)	0.041*
No													
Yes	96 (53.93)	53 (59.55)	43 (48.31)		54 (60.67)	42 (47.19)		46 (51.69)	50 (56.18)		55 (61.80)	41 (46.07)	
Alcohol history													
No	71 (39.89)	39 (43.82)	32 (35.96)	0.376	36 (40.45)	35 (39.33)	1.000	36 (40.45)	35 (39.33)	1.000	37 (41.57)	34 (38.20)	0.750
Yes	107 (60.11)	50 (56.18)	57 (64.04)		53 (59.55)	54 (60.67)		53 (59.55)	54 (60.67)		52 (58.43)	55 (61.80)	
History of diabetes	124 (69.66)	59 (66.29)	65 (73.03)	0.688	68 (76.40)	56 (62.92)	0.038*	67 (75.28)	58 (65.17)	0.075	59 (66.29)	65 (73.03)	0.346
No													
Yes	54 (30.34)	30 (33.71)	24 (26.97)		21 (23.60)	33 (37.08)		22 (24.72)	31 (34.83)		30 (33.71)	24 (26.97)	
History of chronic pancreatitis	150 (84.27)	72 (80.90)	74 (83.15)	1.000	70 (78.65)	77 (86.52)	0.076	72 (80.90)	75 (84.27)	0.893	76 (85.39)	71 (79.78)	0.507
No													
Yes	28 (15.73)	17 (19.10)	15 (16.85)		19 (21.35)	12 (13.48)		17 (19.10)	14 (15.73)		13 (14.61)	18 (20.22)	
Family history of cancer	81 (45.51)	43 (48.31)	37 (41.57)	0.408	43 (48.31)	37 (41.57)	0.408	38 (42.70)	42 (47.19)	0.544	42 (47.19)	38 (42.70)	0.621
No													
Yes	97 (54.49)	46 (51.59)	52 (58.43)		46 (51.69)	52 (59.43)		51 (57.30)	47 (52.81)		47 (52.81)	51 (57.30)	
Anatomic neoplasm subdivision	138 (77.53)	73 (82.02)	65 (73.03)	0.209	66 (74.16)	72 (80.90)	0.369	67 (75.28)	71 (79.78)	0.590	71 (79.78)	67 (75.28)	0.590
Head of Pancreas	40 (22.47)	16 (17.98)	24 (26.97)		23 (25.84)	17 (19.10)		22 (24.72)	18 (20.22)		18 (20.22)	22 (24.72)	
Other													
Primary therapy outcome	58 (32.58)	34 (38.2)	25 (28.09)	0.037*	34 (38.20)	25 (28.09)	0.371	29 (32.58)	30 (33.71)	0.200	33 (37.08)	26 (29.21)	0.548
PD													
SD	19 (10.67)	5 (5.62)	14 (15.73)		9 (10.11)	9 (10.11)		11 (12.36)	7 (7.87)		6 (6.74)	12 (13.48)	
PR	20 (11.24)	8 (8.99)	11 (12.36)		9 (10.11)	10 (11.24)		7 (7.87)	13 (14.61)		9 (10.11)	11 (12.36)	
CR	81 (45.51)	42 (47.19)	39 (43.82)		37 (41.57)	45 (50.56)		42 (47.19)	39 (43.82)		41 (46.07)	40 (44.94)	
Radiation therapy	126 (70.79)	68 (76.40)	58 (65.17)	0.108	66 (74.16)	60 (67.42)	0.365	59 (66.29)	67 (75.28)	0.298	65 (73.03)	61 (68.54)	0.620
No													
Yes	52 (29.21)	21 (23.60)	31 (34.83)		23 (25.84)	29 (32.58)		30 (33.71)	22 (24.72)		24 (26.97)	28 (31.46)	
Residual tumor	112 (62.92)	55 (61.80)	58 (65.17)	0.265	55 (61.80)	58 (65.17)	0.914	51 (57.30)	61 (68.54)	0.378	56 (62.92)	57 (64.04)	0.915
R0													
R1	57 (32.02)	32 (35.96)	25 (28.09)		28 (31.46)	28 (31.46)		32 (35.96)	25 (28.09)		30 (33.71)	28 (31.46)	
R2	9 (5.06)	2 (2.25)	6 (6.74)		6 (6.74)	3 (3.37)		6 (6.74)	3 (3.37)		3 (3.37)	4 (4.49)	
Histologic grade	31 (17.42)	12 (13.48)	19 (21.35)	0.019*	11 (12.36)	21 (23.60)	0.048*	14 (15.73)	17 (19.10)	0.938	12 (13.48)	20 (22.47)	0.080
G1													
G2	96 (53.93)	45 (50.56)	52 (58.43)		48 (53.93)	48 (53.93)		49 (55.06)	48 (53.93)		47 (52.81)	49 (55.06)	
G3	49 (27.53)	32 (35.96)	16 (17.98)		30 (33.71)	18 (20.22)		25 (28.09)	23 (25.84)		30 (33.71)	18 (20.22)	
G4	2 (1.12)	0 (0.00)	2 (2.25)		0 (0.00)	2 (2.25)		1 (1.12)	1 (1.12)		0 (0.00)	2 (2.25)	
Pathologic stage	22 (12.36)	9 (10.11)	13 (14.61)	0.171	5 (5.62)	17 (19.10)	0.004*	13 (14.61)	9 (10.11)	0.252	8 (8.99)	14 (15.73)	0.261
Stage I													
Stage II	147 (82.58)	76 (85.39)	72 (80.90)		80 (89.89)	68 (76.40)		73 (82.02)	75 (84.27)		79 (88.76)	69 (77.53)	
Stage III	3 (1.69)	0 (0.00)	3 (3.37)		0 (0.00)	3 (3.37)		0 (0.00)	3 (3.37)		1 (1.12)	2 (2.25)	
Stage IV	6 (3.37)	4 (4.49)	1 (1.12)		4 (4.49)	1 (1.12)		3 (3.37)	2 (2.25)		1 (1.12)	4 (4.49)	
T stage	7 (3.93)	4 (4.49)	3 (3.37)	0.143	3 (3.37)	4 (4.49)	0.020*	3 (3.37)	4 (4.49)	0.343	4 (4.49)	3 (3.37)	0.893
T1													
T2	25 (14.04)	9 (10.11)	16 (17.98)		7 (7.87)	18 (20.22)		14 (15.73)	11 (12.36)		11 (12.36)	13 (14.61)	
T3	143 (80.34)	76 (85.39)	67 (75.28)		79 (88.76)	64 (71.91)		72 (80.90)	71 (79.78)		73 (82.02)	71 (79.78)	
T4	3 (1.69)	0 (0.00)	3 (3.37)		0 (0.00)	3 (3.37)		0 (0.00)	3 (3.37)		1 (1.12)	2 (2.25)	
N stage	52 (29.21)	26 (29.21)	26 (29.21)	1.000	26 (29.21)	27 (30.34)	1.000	23 (25.84)	29 (32.58)	0.429	26 (29.21)	26 (29.21)	1.000
N0													
N1	126 (70.79)	63 (70.79)	63 (70.79)		63 (70.79)	62 (69.66)		66 (74.16)	60 (67.42)		63 (70.79)	63 (70.79)	
M stage	126 (70.79)	65 (73.03)	61 (68.54)	0.396	65 (73.03)	62 (69.66)	0.386	63 (70.79)	64 (71.91)	1.000	63 (70.79)	63 (70.79)	0.366
M0													
M1	52 (29.21)	24 (26.97)	28 (31.46)		24 (26.97)	27 (30.34)		26 (29.21)	25 (28.09)		26 (29.21)	26 (29.21)	

### Enrichment Analysis of the KDM5 Family and 800 Co-Expressed Genes in Patients With PAAD

**FIGURE 4:**
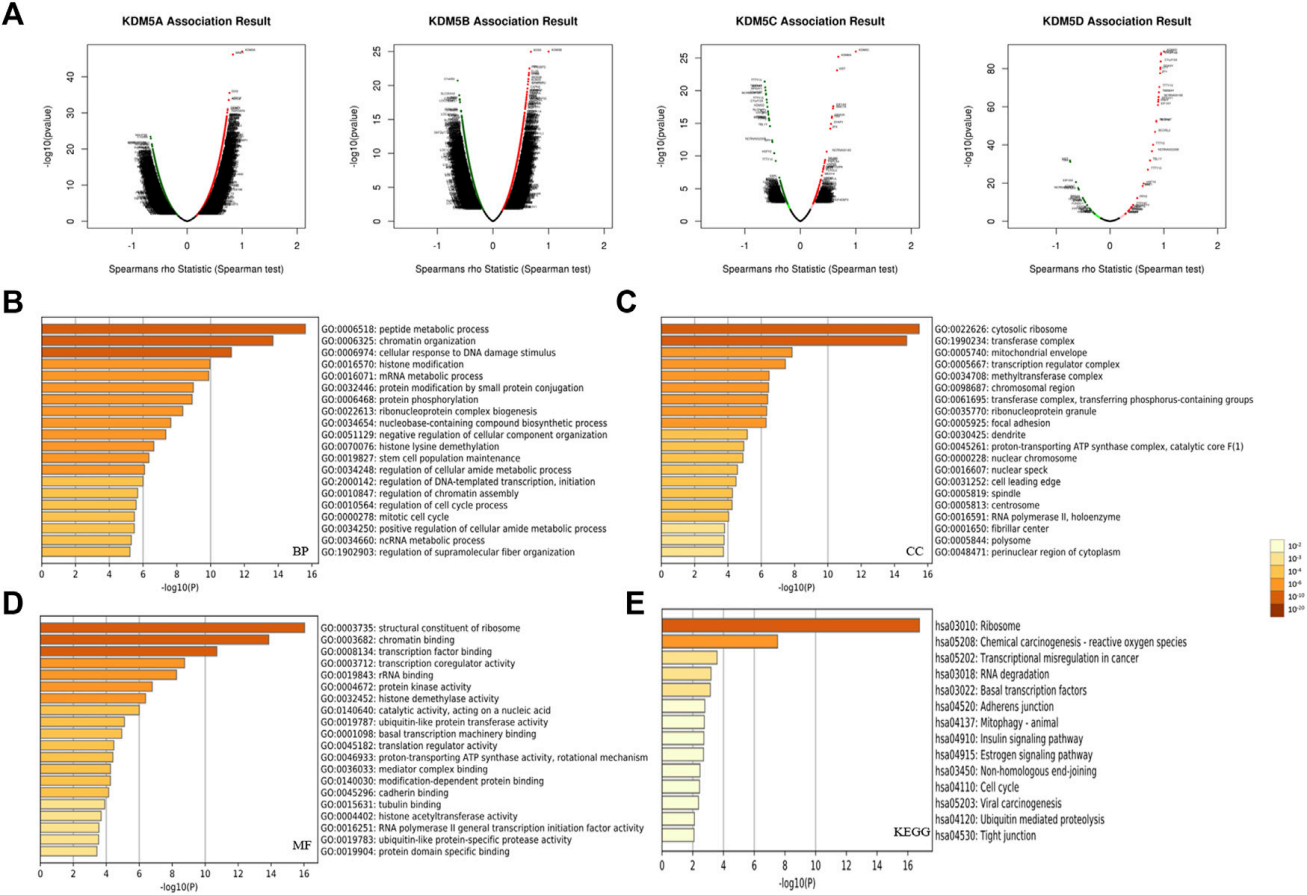
Functional enrichment analysis of the KDM5 family and its 800 co-expressed genes in patients with PAAD. **(A)** A volcano plot of the KDM5 family and its co-expressed genes in pancreatic cancer; **(B)** The GO enrichment of the BP terms of 800 co-expressed genes; **(C)** The GO enrichment of the CC terms of 800 co-expressed genes; **(D)** The GO enrichment of the MF terms of 800 co-expressed genes; **(E)** The KEGG enrichment of the 800 co-expressed genes.

**FIGURE 5:**
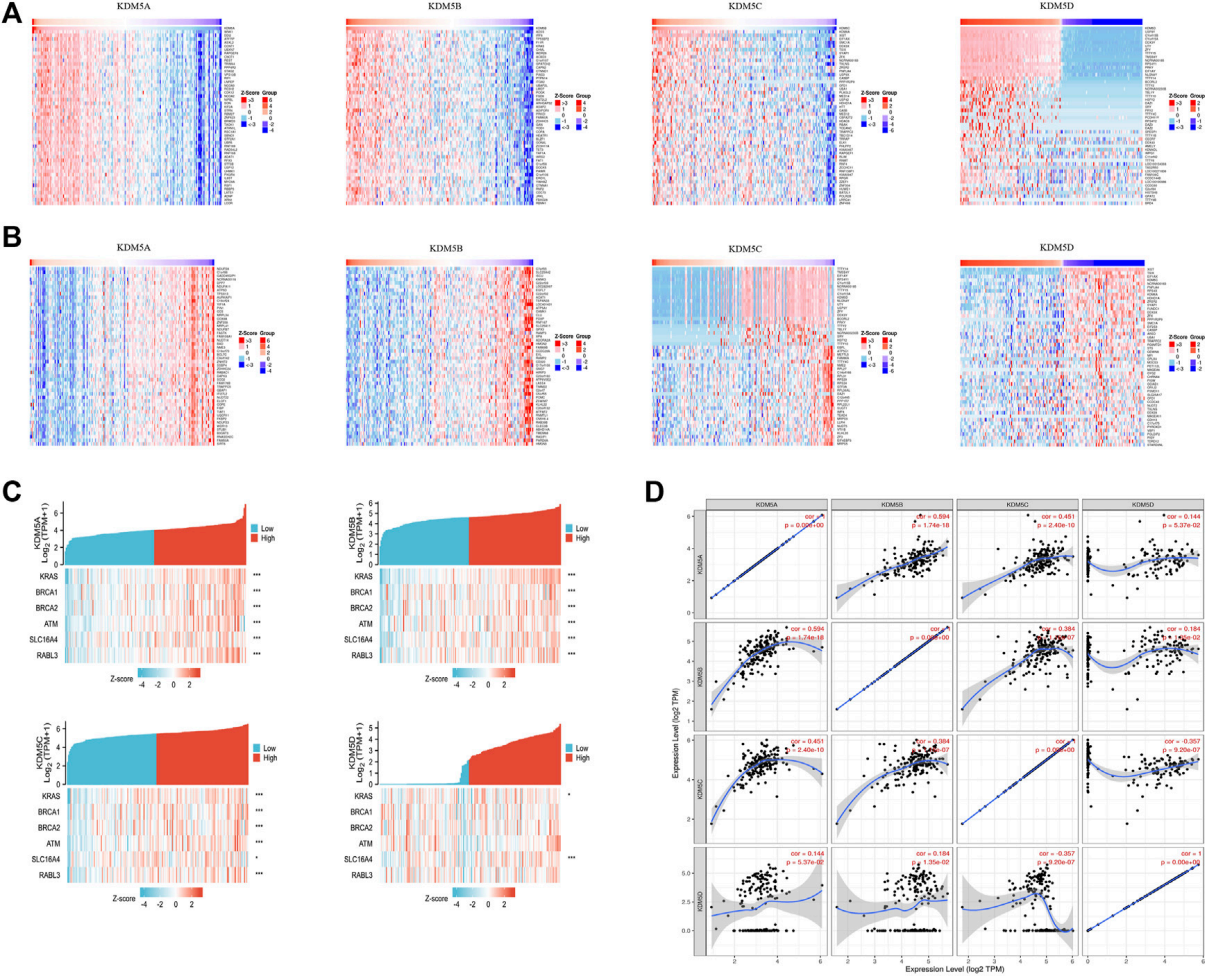
The top 50 positively correlated genes and top 50 negatively correlated genes of the KDM5 family genes in patients with PAAD; Co-expression correlations of KDM5 family members and pathogenic genes of PAAD. **(A)** The top 50 genes with a positive correlation with KDM5 family genes are visualized in a heatmap; **(B)** The top 50 genes with a negative correlation with KDM5 family genes are visualized in a heatmap; **(C)** The expression of KDM5A/B/C is positively correlated with the expression of KRAS, BRCA1, BRCA2, ATM, SLC16A4 and RABL3. *, *p* < 0.05; **, *p* < 0.01; ***, *p* < 0.001; **(D)** The expression of each KDM5 family member is significantly positively correlated.

To explore the interaction between KDM5 family genes and their co-expressed genes in pancreatic cancer, 800 co-expressed genes of the KDM5 family in pancreatic cancer were obtained from the LinkedOmics database (Supplementary Material S1), and a volcano plot of the KDM5 family and co-expressed genes in pancreatic cancer was drawn (Figure 4A). Then, we analyzed the GO and KEGG pathway terms of these 800 genes using the Metascape database. To study the functional mechanism of the KDM5 family in the occurrence and development of pancreatic cancer, GO analysis was performed. The results showed significant enrichment of the BP terms “peptide metabolic process”, “chromatin organization”, and “cellular response to DNA damage stimulus” (Figure 4B). The enriched CC terms mainly included “cytosolic ribosome”, “transferase complex” and “mitochondrial envelope” (Figure 4C). The enriched MF terms mainly included “structural constituent of ribosome”, “chromatin binding”, and “transcription factor binding” (Figure 4D). The KEGG pathway analysis showed that the target genes were mainly associated with the terms “ribosome”, “chemical carcinogenesis—reactive oxygen species”, and “transcriptional misregulation in cancer” (Figure 4E). To further analyze the mechanisms of the KDM5 family and its coexpressed genes, the top 50 genes with a positive correlation (Figure 5A) and the top 50 genes with a negative correlation (Figure 5B) with KDM5 family genes were visualized in a heatmap. GeneCards was used to annotate the top 10 genes positively related to the expression of KDM5A/B/C and the first 10 genes negatively related to the expression of KDM5D. The results showed that the functions of related genes included transcriptional regulation (DDI2, ASXL2, CCNT1, CNOT1, REST, TRIM44, IRF6, KDM6A, EIF1AX, DDX3X, TSIX, ZFX, TXLNG, KRAS, GPATCH2, and ZRSR2), protein modification (WNK1, UBXN7, RAPGEF6, CHML, WDR26, and SYAP1), DNA damage repair (SMC1A), cell cycle regulation (TP53BP2), and cell migration regulation (F11R). Then, the top 10 genes negatively related to the expression of KDM5A/B/C and the top 10 genes positively related to the expression of KDM5D were functionally annotated. The results showed that the functions of the genes included transcriptional regulation (DDX3Y, ZFY, and RPS4Y1), protein modification (USP9Y and UTY), translation regulation (EIF1AY and RPS4Y1), cell cycle regulation (GADD45GIP1), cell migration regulation (NLGN4Y), cell proliferation regulation (TMSB4Y, TP53I13, EGFL7, and TSPAN33) and apoptosis regulation (DPP7). Notably, our analysis showed that the KRAS gene was positively related to the expression of KDM5B (Figure 5A). KRAS can induce transcriptional silencing of tumor suppressor genes, and its mutations produce modified proteins that drive the occurrence and development of pancreatic cancer (Asimgil et al., 2022). This correlation further suggested that the KDM5 family plays a unique role in malignant tumors driven by KRAS mutations, including pancreatic cancer. At the same time, this important finding inspired us to analyze the correlation between the expression of KDM5 family genes and other pathogenic genes in pancreatic cancer. We used the R (version 3.6.3) to analyze the TCGA PAAD data. The results showed that the expression level of KDM5A/B/C was positively correlated with the expression of KRAS, BRCA1, BRCA2, ATM, SLC16A4, and RABL3. We speculated that although some of the genes known to be pathogenic in pancreatic cancer were not in the top 50 genes positively correlated with the expression of KDM5 family genes, they may still have a co-expression relationship with the KDM5 family. Furthermore, it is suggested that the KDM5 family may play an important role in the pathogenesis of pancreatic cancer. Finally, we used the TIMER database to analyze the relationships among the members of the KDM5 family, and the results showed that the expression of each member was significantly positively correlated: the correlation between KDM5A and KDM5B was the strongest (cor = 0.594) (Figure 5D). According to the above results, we speculated that members of the KDM5 family can cooperate with a variety of pathogenic genes, transcriptional regulatory factors, protein-modifying factors, DNA damage repair factors, cell cycle regulatory factors, and cell migration regulatory factors in pancreatic cancer, including factors related to the terms “chemical carcinogenesis—reactive oxygen species”, and “transcriptional misregulation in cancer”, to participate in the occurrence and development of pancreatic cancer.

### Gene Enrichment Analysis of KDM5 Family Genes and PPI Network Members in PAAD

**FIGURE 6:**
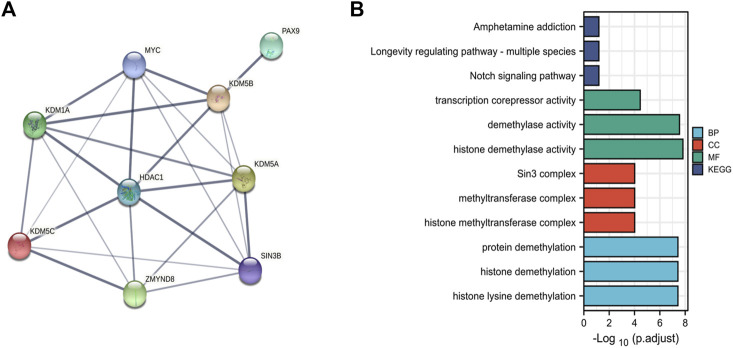
PPI and functional enrichment analysis of the KDM5 family and related genes in patients with PAAD. **(A)** The PPI network associated with the KDM5 family in pancreatic cancer; **(B)** The GO and KEGG pathway terms of the 9 genes with the strongest interaction with KDM5 family proteins.).

To explore the PPIs between KDM5 family genes and related genes in pancreatic cancer, we analyzed the protein expression of members of the KDM5 family and related genes using the STRING database (Supplementary Material S2). The nine genes with the strongest protein-protein interaction (PPI) with KDM5 family members were determined. We analyzed the PPI network related to the KDM5 family expression in pancreatic cancer. The PPI network included 9 gene nodes and 24 edges (Figure 6A). The results showed that the protein expression of KDM5A/B/C was closely related to that of HDAC1. The protein encoded by HDAC1 can deacetylate part of the lysine residue of histone H3 and plays an important role in transcriptional regulation and cell proliferation (Cai et al., 2000). The proteins encoded by the KDM5 family play a role in the regulation of gene expression through the specific demethylation of histone H3 lysine 4 (Zhang et al., 2014). Both proteins act on histone H3 lysines, suggesting that they may have a synergistic effect in the regulation of gene expression. Next, we used the R (version 3.6.3) to analyze the GO and KEGG pathway terms of the 9 genes with the strongest interaction with KDM5 family proteins (Figure 6B). The results of the GO analysis showed significant enrichment of the BP terms “histone lysine demethylation”, “histone demethylation”, and “protein demethylation”. The significantly enriched CC terms mainly included “histone methyltransferase complex”, “methyltransferase complex”, and “Sin3 complex”. The significantly enriched MF terms mainly included “histone demethylase activity”, “demethylase activity”, and “transcription corepressor activity”. The KEGG pathway analysis showed that the target genes were mainly associated with the terms “longevity-regulating pathway”, “Notch signaling pathway”, and “amphetamine addiction”. The above results show that KDM5 family proteins may have strong interactions with HDAC1 and other proteins, modify the lysines of histone H3, and act on many pathways, including the “longevity-regulating pathway” and “Notch signaling pathway”, which play a regulatory role in the proliferation of pancreatic cancer cells.

### Relationship Between the KDM5 Family and Tumor-Infiltrating Immune Cells and Immune Molecules in Patients With PAAD

**FIGURE 7:**
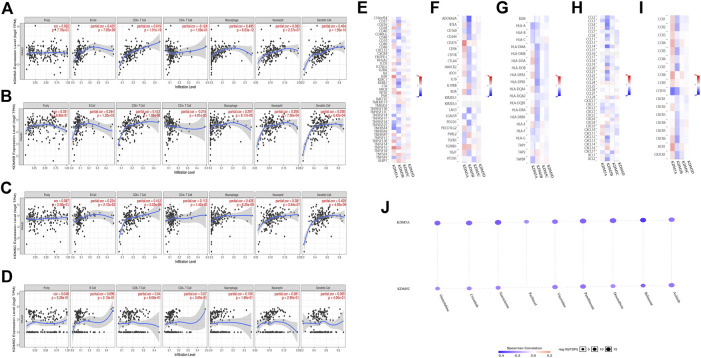
Relationships between the expression levels of KDM5 family members and tumor-infiltrating immune cells, immune molecules, and sensitivity to pancreatic cancer-targeting and chemotherapeutic drugs. **(A–C)** Upregulation of KDM5A/B/C expression is associated with increased infiltration of B cells, CD8^+^ T cells, macrophages, neutrophils, dendritic cells, and other infiltrating lymphocytes; **(D)** Upregulation of KDM5D expression is not associated with increased infiltration of B cells, CD8^+^ T cells, macrophages, neutrophils, dendritic cells and other infiltrating lymphocytes; **(E–I)** The correlation between KDM5 family expression and immunostimulatory molecules, immunosuppressive molecules, MHC molecule, chemokines, and chemokine receptors in pancreatic cancer; **(J)** The expression levels of KDM5A and KDM5C are negatively correlated with sensitivity to many pancreatic cancer-targeting and chemotherapeutic drugs, including axitinib, and gemcitabine.

The immune system is a complex system in which immune cells both act as the first line of defense against a variety of pathogens and provide surveillance by identifying and destroying latent cancer cells. However, in some cases, the immune system can help tumor cells escape immune control (Shalapour and Karin, 2015). Tumor-infiltrating lymphocytes are a unique kind of lymphocytes that infiltrate the tumor microenvironment by detecting cancer antigens and releasing proinflammatory cells. We used the TIMER database to further explore the relationship between the expression of KDM5 family genes and the level of infiltrating lymphocytes. The results showed that upregulation of KDM5A/B/C expression was associated with increased infiltration of B cells, CD8^+^ T cells, macrophages, neutrophils, dendritic cells and other infiltrating lymphocytes (Figures 7A–C) and the upregulation of KDM5D expression was not associated with increased infiltration of B cells, CD8^+^ T cells, macrophages, neutrophils, dendritic cells and other infiltrating lymphocytes (Figure 7D). Next, we analyzed the correlation between KDM5 family expression and immunostimulatory molecules (Figure 7E), immunosuppressive molecules (Figure 7F), MHC molecules (Figure 7G), chemokines (Figure 7H), and chemokine receptors (Figure 7I) in pancreatic cancer using the TISIDB database. The results showed that upregulated expression of KDM5A/B/C was associated with an increase in the expression of immunostimulatory molecules such as NT5E, TNFSF4, and TNFSF15, immunosuppressive molecules such as CD274 and TGFBR1, MHC molecules such as TAP2, chemokines such as CCL24, and chemokine receptors such as CCR8 and CCR9 (Figures 7E–I), which provides important information for predicting potential therapeutic targets. Finally, we used GSCALiteonlinetool to analyze the relationship between the expression of KDM5 family genes and sensitivity to current immune or targeted therapies for pancreatic cancer (Figure 7J). The results showed that the expression levels of KDM5A and KDM5C were negatively correlated with sensitivity to many pancreatic cancer-targeting or chemotherapeutic drugs, including axitinib and gemcitabine. Thus, the KDM5 family might represent a new target for predicting drug sensitivity and for developing multitarget combined therapy for pancreatic cancer.

## Discussion

Unlike other histone-modifying enzymes, members of the KDM5 family of histone lysine demethylases contain an ARID domain and thus can recognize the specific DNA sequence in targets; this feature is also an important marker to distinguish the KDM5 family from other histone lysine demethylase families (Blair et al., 2011). In recent years, an increased understanding of the carcinogenic role of the KDM5 family has been gained, and related experimental studies have found a potential cancer-promoting role of the KDM5 family in a variety of malignant tumors, including breast cancer, lung cancer, and prostate cancer (Hou et al., 2012; Stein et al., 2014; Li et al., 2016; Feng et al., 2017; Gong et al., 2018; Kuo et al., 2018; Oser et al., 2019; Du et al., 2020; Xue et al., 2020; Gu and Chu, 2021). However, research on the role and mechanism of the KDM5 family in the occurrence and development of pancreatic cancer is still lacking, and specific bioinformatics analyses have not been carried out. This study is the first to analyze the expression, gene mutation, relationship with immune cell infiltration and prognostic role of the KDM5 family in pancreatic cancer. We employed multiple public databases to reveal that the expression of KDM5A, KDM5B, and KDM5C (all members of the KDM5 family) is significantly increased in pancreatic cancer. In contrast, the expression of KDM5D was significantly decreased, and the expression level KDM5 family members was closely related to tumor stage, tumor grade, treatment efficacy, and other clinicopathological factors, suggesting that KDM5 family members have significant prognostic and diagnostic value and can be used as potential biomarkers for the diagnosis and prognostication of pancreatic cancer. In addition, we revealed a possible mechanism by which the KDM5 family participates in the occurrence and development of pancreatic cancer and its relationship with the tumor immune response, providing potential targets for multitarget combined therapy of pancreatic cancer, and important clinical significance.

Studies have shown that KDM5A promotes the resistance of breast cancer cells to clinical drugs such as trastuzumab and erlotinib by blocking the regulation of p21 and BCL2-antagonist/killer 1 (Bak1) (Hou et al., 2012; Choi et al., 2018). Similarly, our study revealed that the expression of KDM5A can also reduce the sensitivity of pancreatic cancer cells to a variety of targeted and chemotherapeutic drugs (such as gemcitabine and paclitaxel). Because KDM5A can lead to tumor cell chemotherapy resistance in a variety of cancers, we urgently need to further study the mechanism of drug resistance to develop new therapeutic targets for pancreatic cancer. In addition, compared with that in normal prostate tissue, the expression of KDM5A in prostate cancer tissue was upregulated (Vieira et al., 2013). This overexpression significantly reduced the methylation level of H3K4, which in turn reduced the expression level of the KLF4 and E-cadherin genes, which suppress tumor cell proliferation, and made prostate cancer more invasive (Huang et al., 2011). KDM5A can also promote the progression of prostate cancer through the KDM5A/miRNA-495/YTHDF2/m6A-MOB3B axis (Du et al., 2020). This study revealed a possible synergistic effect of KDM5A and m6A regulators in the pathogenesis of prostate cancer. In addition, KDM5A can also promote the occurrence of small-cell lung cancer by inhibiting the target genes NOTCH1 and NOTCH2 (Oser et al., 2019). The results of KEGG pathway analysis of the genes with the strongest interaction with KDM5 family proteins in this study also suggest that the family may promote the occurrence and development of pancreatic cancer through the NOTCH pathway. Intriguingly, the GO and KEGG analyses of the KDM5 family and its 800 coexpressed genes in this study suggest that they play a role in many biological processes, such as “cell cycle regulation”, “chromatin binding”, “transcription factor binding” and “transcriptional disorders in cancer”, which is consistent with the finding that overexpression of KDM5A promotes the proliferation, invasion, and metastasis of many kinds of tumors. The KDM5A family likely plays a key role in regulating the proliferation of pancreatic cancer cells and inducing chemotherapy resistance.

KDM5B is a transcriptional inhibitor that specifically demethylates histone H3 lysine 4 (H3K4), putting it in a state of transcriptional inactivity (Benevolenskaya, 2007). Related studies have shown that KDM5B inhibits the expression of PTEN at the transcriptional level through H3K4 demethylation, thus inhibiting phosphorylated PI3K and AKT and increasing the proliferation, migration and invasion of hepatocellular carcinoma cells *in vivo* and *in vitro* (Tang et al., 2015). In addition, in syngeneic mouse breast tumor models and xenotransplantation models, KDM5B knockout leads to upregulation of tumor suppressor genes such as BRCA1, CAV1, and HOXA5 (Yamane et al., 2007) and increased H3K4 methylation in the chromatin regions of these target genes (Rasmussen and Staller, 2014). KDM5B is also related to chemotherapeutic drug resistance in NSCLC. Related experiments have shown that knockout of the KDM5B gene enhances the death of NSCLC cells induced by cisplatin and doxorubicin, suggesting that KDM5B may promote the invasiveness of NSCLC cells through epigenetic regulation of epithelial-mesenchymal transformation (EMT) regulatory factors such as vimentin, snail, and E-cadherin and upregulation of multipotent transcription factors such as OCT4, SOX2, KLF4, and c-Myc (Kristensen et al., 2012; Rasmussen and Staller, 2014; Yamamoto et al., 2014). These studies have revealed that KDM5B may be a marker of malignant tumor progression and a potential therapeutic target.

There is an interaction between KDM5C and histone deacetylases (HDACs), which have been successfully targeted in cancer therapy (Huang et al., 2011), consistent with the results of our protein interaction analysis. In addition, some studies have shown that the success of HDAC inhibitors is closely related to their interaction with KDM5C. HDACs usually act on many different histone residues, while the catalytic activity of KDM5C is limited to specific histone residues (Huang et al., 2011). This suggests that KDM5C inhibitors may have more specific biological effects and are more specific anticancer drugs than HDAC inhibitors. In addition, Johannes Stein et al. found that KDM5C gene knockout leads to growth retardation of prostate cancer cells *in vitro* and induces the regulation of several proliferation-related genes. This finding implies that KDM5C is an ideal drug target for prostate cancer (Stein et al., 2014). Recent studies have found that KDM5C is also highly expressed in ER-positive primary gastric cancer, regulated by ER and HIF1, and can significantly promote the proliferation, migration and invasion of gastric cancer cells (Xu et al., 2017). This study provides additional strong evidence for the role of KDM5C in promoting cancer. Therefore, we speculate that KDM5C may also play the role of an oncogene in pancreatic cancer and expect it to become a new target for tumor therapy.

KDM5D is a male-specific protein that inhibits the expression of genes associated with cell invasion (Li et al., 2016). There is growing evidence that EMT is necessary for tumor metastasis, and KDM5D gene knockout increases the expression of key EMT regulators (such as N-cadherin and Slug) (Li et al., 2016). In addition, related studies have shown that the expression of ETV4 is significantly increased in the process of gastric cancer cell proliferation and is closely related to lymph node metastasis, distant metastasis, and poor prognosis of gastric cancer patients, while KDM5D can downregulate the expression of ETV4 (Cai et al., 2020). In addition, overexpression of KDM5D can increase the sensitivity of cancer patients to ATR inhibitors or cell cycle inhibitors (Schäfer et al., 2021). These studies suggest that decreased expression of KDM5D may be an important reason for the occurrence of many kinds of cancers and tumor drug resistance.

Another key finding of this study is that the expression levels of KDM5 family members are related to the infiltration of many kinds of infiltrating lymphocytes and the expression level of immune molecules in pancreatic cancer. The immune system is a complex system (Saab et al., 2020). In addition to acting as the first line of defense against a variety of pathogens, immune cells can also provide surveillance by identifying and destroying latent cancer cells. However, in some cases, the immune system can help tumor cells escape immune control (Shalapour and Karin, 2015). Tumor-infiltrating lymphocytes are a unique kind of lymphocytes that infiltrate the tumor microenvironment by detecting cancer antigens and releasing proinflammatory and immune molecules that are important substances that regulate the immune function of the body (Lee et al., 2016). Upregulation of KDM5A/B/C expression was associated with increased infiltration of B cells, CD8^+^ T cells, macrophages, neutrophils, dendritic cells, and other infiltrating lymphocytes and increased expression of immunostimulatory molecules such as NT5E, TNFSF4, and TNFSF15, immunosuppressive molecules such as CD274 and TGFBR1, MHC molecules such as TAP2, and chemokine receptors such as CCL24, CCR8, and CCR9. These findings prove that the KDM5 family is closely related to immune function in pancreatic cancer, which provides important information for predicting potential therapeutic targets. Intriguingly, we found that overexpression of KDM5A/C was associated with reduced sensitivity of pancreatic cancer cells to a variety of pancreatic cancer-targeting and chemotherapeutic drugs, such as axitinib and gemcitabine. This discovery provides strong evidence for the study of KDM5A and KDM5C as targets for new pancreatic cancer-targeting and chemotherapeutic drugs. However, this study has some limitations. For example, the number of databases included in this study is somewhat inadequate. In addition, this study is only a bioinformatics analysis of the function of KDM5 family in PAAD. Future basic research may further confirm the tumor-promoting or tumor-suppressing role of the KDM5 family in PAAD.

## Conclusion

In summary, our bioinformatics analysis of the KDM5 family and the pathogenesis of pancreatic cancer found that KDM5 family members can be used as prognostic markers and new therapeutic targets for patients with pancreatic cancer. However, relevant experimental studies *in vivo* and *in vitro* are urgently needed. Importantly, the increased understanding of the pathogenic mechanism by which this family participates in pancreatic cancer is expected to significantly improve the efficacy of multimodal comprehensive treatment of pancreatic cancer.

## Abbreviations

PAAD, Pancreatic adenocarcinoma; FOLFIRINOX:5-fluorouracil, leucovorin, irinotecan, and oxaliplatin; GNP, Gemcitabine and naphthaclitaxel; TCGA, The cancer genome atlas; GTEx, Genotype-tissue expression; OS, Overall survival; RFS, Relapse free survival; DFS, Disease-free survival; GO, Gene ontology; KEGG, Kyoto encyclopedia of genes and genomes; DNA, Deoxyribonucleic acid; RNA, Ribonucleic acid; NSCLC, Non-small cell lung cancer; EMT, Epithelial-mesenchymal transformation; HDAC, Histone deacetylase family; PPI, Protein-protein interaction; BP, Biological process; CC, Cellular composition; MF, Molecular function; BRCA, Breast invasive carcinoma; STAD, Stomach adenocarcinoma; ACC, Adrenocortical carcinoma; SKCM, Skin cutaneous melanoma; PRAD, Prostate adenocarcinoma; Bak1, BCL2-antagonist/killer 1.

## Data Availability

The original contributions presented in the study are included in the article/Supplementary Material, further inquiries can be directed to the corresponding author.

## Supplementary Material

Supplementary Figure S1 Prognostic characteristics of patients with and without gene mutations of the KDM5 family. (A)OS; B(FPS).

Supplementary Table S1 800 coexpressed genes of the KDM5 family in PAAD.

Supplementary Table S2 Protein expression of KDM5 family members and related genes.

## Abbreviations

PAAD, Pancreatic adenocarcinoma; FOLFIRINOX:5-fluorouracil, leucovorin, irinotecan, and oxaliplatin; GNP, Gemcitabine and naphthaclitaxel; TCGA, The cancer genome atlas; GTEx, Genotype-tissue expression; OS, Overall survival; RFS, Relapse free survival; DFS, Disease-free survival; GO, Gene ontology; KEGG, Kyoto encyclopedia of genes and genomes; DNA, Deoxyribonucleic acid; RNA, Ribonucleic acid; NSCLC, Non-small cell lung cancer; EMT, Epithelial-mesenchymal transformation; HDAC, Histone deacetylase family; PPI, Protein-protein interaction; BP, Biological process; CC, Cellular composition; MF, Molecular function; BRCA, Breast invasive carcinoma; STAD, Stomach adenocarcinoma; ACC, Adrenocortical carcinoma; SKCM, Skin cutaneous melanoma; PRAD, Prostate adenocarcinoma; Bak1, BCL2-antagonist/killer 1.
