# Supplementary figures and images for: Expression, Prognostic Value, and Functional Mechanism of the KDM5 Family in Pancreatic Cancer
Source: Front Cell Dev Biol. 2022 Apr 13;10:887385. doi: 10.3389/fcell.2022.887385 (PMC9043291; doi:10.3389/fcell.2022.887385)

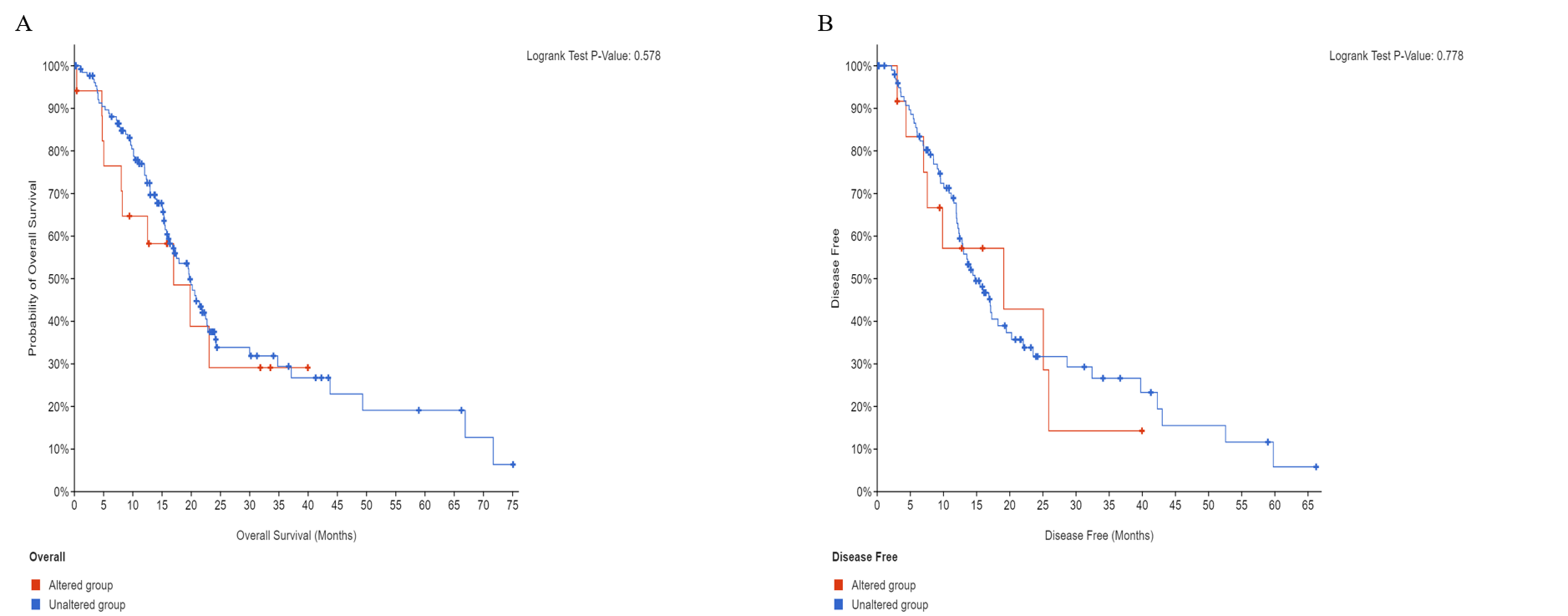

Supplement: Supplementary file 3 [file Image1.TIF]
